# Loseolamycins: A Group of New Bioactive Alkylresorcinols Produced after Heterologous Expression of a Type III PKS from *Micromonospora endolithica*

**DOI:** 10.3390/molecules25204594

**Published:** 2020-10-09

**Authors:** Constanze Lasch, Nils Gummerlich, Maksym Myronovskyi, Anja Palusczak, Josef Zapp, Andriy Luzhetskyy

**Affiliations:** 1 Pharmaceutical Biotechnology, Saarland University, 66123 Saarbruecken, Germany; constanze.lasch@uni-saarland.de (C.L.); nils.gummerlich@gmx.de (N.G.); maksym.myronovskyi@uni-saarland.de (M.M.); anja.palusczak@uni-saarland.de (A.P.); 2 Pharmaceutical Biology, Saarland University, 66123 Saarbruecken, Germany; j.zapp@mx.uni-saarland.de; 3 Helmholtz Institute for Pharmaceutical Research Saarland, 66123 Saarbruecken, Germany

**Keywords:** alkylresorcinol, heterologous expression, *Micromonospora endolithica*, *Streptomyces albus* Del14, type III polyketide synthase, biosynthetic gene

## Abstract

Natural products are a valuable source of biologically active compounds with potential applications in medicine and agriculture. Unprecedented scaffold diversity of natural products and biocatalysts from their biosynthetic pathways are of fundamental importance. Heterologous expression and refactoring of natural product biosynthetic pathways are generally regarded as a promising approach to discover new secondary metabolites of microbial origin. Here, we present the identification of a new group of alkylresorcinols after transcriptional activation and heterologous expression of the type III polyketide synthase of *Micromonospora endolithica*. The most abundant compounds loseolamycins A1 and A2 have been purified and their structures were elucidated by NMR. Loseolamycins contain an unusual branched hydroxylated aliphatic chain which is provided by the host metabolism and is incorporated as a starter fatty acid unit. The isolated loseolamycins show activity against gram-positive bacteria and inhibit the growth of the monocot weed *Agrostis stolonifera* in a germination assay. The biosynthetic pathway leading to the production of loseolamycins is proposed in this paper.

## 1. Introduction

In the last century, actinobacteria have proven to be a reliable source of natural products with various biological activities. Many of these compounds have found commercial application in medicine and agriculture as antibiotics, anticancer drugs, immunosuppressives, anthelmintics, insecticides, etc. [1,2]. Besides their interesting biological activities, natural products are of great fundamental interest. Unprecedented structural entities of natural products and biosynthetic routes leading to their production are the object of study of numerous research groups [3,4,5]. For the above mentioned reasons, the discovery of new natural products still remains an important research topic.

The discovery of structurally new secondary metabolites of microbial origin has become a tough task. The classical approach for metabolite discovery which is based on the analysis of crude extracts of naturally occurring strains has lost its efficiency due to, often, the rediscovery of already known compounds. High rediscovery rates have led to the assumption that the potential of actinobacteria as a source of new natural products is exhausted [6]. However, the constantly growing amount of genomic data implies that the potential of actinobacteria to produce secondary metabolites greatly exceeds the number of compounds isolated from the strains. Obviously, only few pathways from several dozens encoded in the genome of each single actinobacterial strain are efficiently expressed under standard laboratory conditions. A great amount of natural product biosynthetic pathways remains silent or is expressed on a very low level in laboratory, preventing the encoded compounds from being discovered. This situation enforced the need for new strategies to uncover the biosynthetic potential inhered in actinobacteria [7,8]. The availability of genome sequencing data as well as of genome-optimized actinobacterial chassis strains led to the development of a new heterologous expression approach for the discovery of new natural products [9]. The genes encoding biosynthetic pathways potentially leading to new compounds are first identified in the genomes with the help of established secondary metabolite genome mining tools. The identified pathways are then cloned and expressed in a panel of optimized heterologous host strains. This technique of heterologous gene expression allows the targeted expression of promising natural products encoding gene clusters and almost completely eliminates the problem of assignment of the identified natural products to the biosynthetic clusters responsible for their production [10]. Optimized levels of biosynthetic precursors and simplified metabolic background of heterologous expression hosts positively affect the production yields of the expressed compounds and simplify their purification for structure elucidation and biological activity studies [11,12]. Due to the mentioned advantages, the heterologous expression approach is regarded as superior to the classical one and is believed to solve the rediscovery problem [10].

In this work, we present the discovery and characterization of structurally new and biologically active compounds. The isolated compounds belong to the alkylresorcinol family of natural products and were given the name loseolamycins. The production of loseolamycins was achieved through the transcriptional activation of the gene encoding a type III polyketide synthase from *Micromonospora endolithica* and its heterologous expression in the cluster-free *Streptomyces albus* Del14 strain. The most abundant products, loseolamycins A1 and A2, were successfully isolated and their structures were solved by NMR. Loseolamycins possess an unusual branched aliphatic chain which is hydroxylated in the one but last position. Individual loseolamycins differ in the length of the chain and its saturation grade. The bioactivity studies revealed antibacterial and herbicidal activities of loseolamycins. The biosynthetic route leading to the production of loseolamycins is discussed in this paper.

## 2. Results and Discussion

### 2.1. Identification and Expression of the Type III PKS Gene

**Figure 1:**
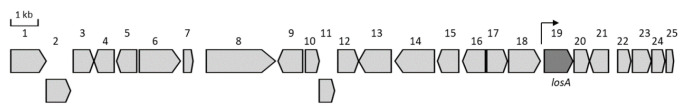
Gene organization of the *M. endolithica* chromosomal fragment cloned in BAC I7. The gene *losA* encoding the putative type III PKS is highlighted in dark grey. The arrow indicates the insertion position of the promoter cassette.

Strains of the class of actinobacteria not belonging to the ubiquitous genus of *Streptomyces* are usually referred to as rare. They are frequently underexplored and represent a remarkable source for new metabolites. In the frame of the project aiming to discover new natural products through heterologous expression, the rare actinomycete *Micromonospora endolithica* has been chosen as a source of secondary metabolite clusters. The putative clusters encoding the production of natural products were identified and annotated in the chromosome of the strain using the genome mining software antiSMASH [13]. To facilitate the heterologous expression of the identified clusters, a genomic library of *M. endolithica* was constructed on an integrative BAC vector. In this study, we focused on gene *LU17765_001730* encoding a putative type III polyketide synthase [14]. From this point on, the gene *LU17765_001730* is designated in the text as *losA*. The BAC I7 containing the *losA* gene was selected from the constructed genomic library. A 24-kb fragment of the *M. endolithica* chromosome is cloned in BAC I7. Besides the gene *losA*, 24 open reading frames were annotated within the cloned fragment (Figure 1, Table 1). In order to express the *losA* gene heterologously, the BAC I7 was transferred into the genome-minimized host *Streptomyces albus* Del14 by conjugation [15]. The obtained exconjugant strain *S. albus* I7 was analyzed for the production of new compounds. For this purpose, the constructed strain *S. albus* I7 harboring the *losA* gene was cultivated in the production medium DNPM. The *S. albus* Del14 strain was used as negative control. LC-MS analysis of the culture extracts did not reveal any new peaks in the culture of the recombinant strain. One of the possible reasons for the failed heterologous expression might be poor expression of the *losA* gene in heterologous environment.

**Figure 2:**
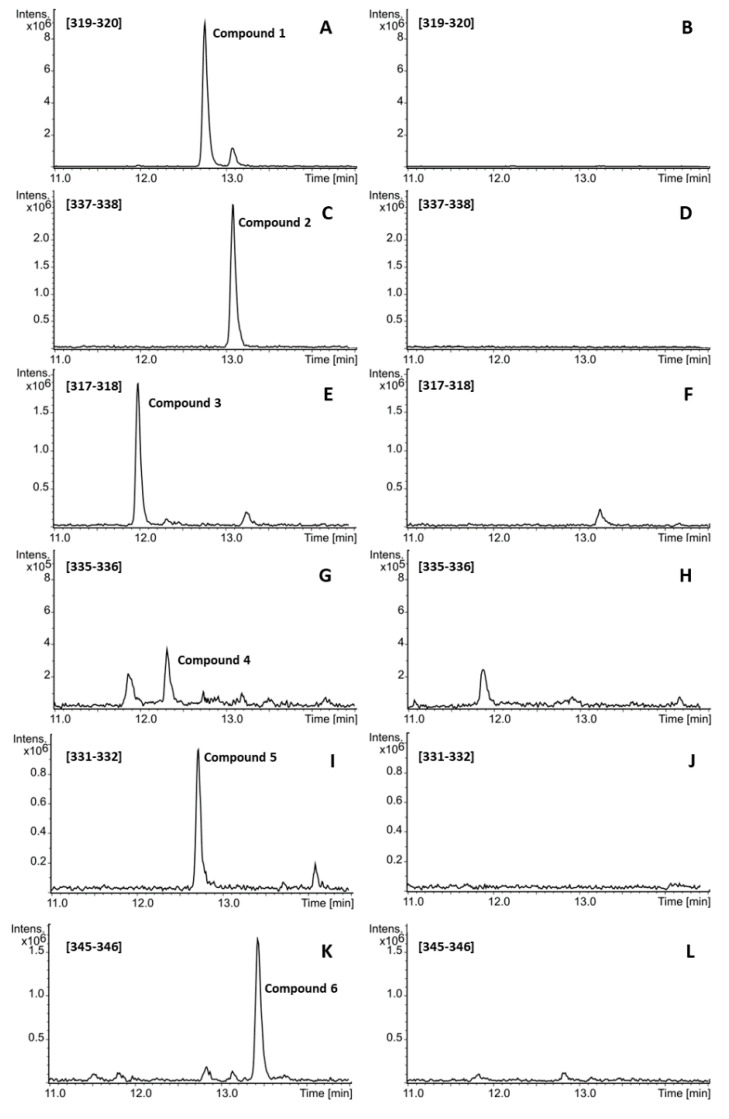
LC-MS analysis of loseolamycin production (compounds **1** to **6**) by the strain *S. albus* I7act. (**A**,**C**,**E**,**G**,**I**,**K**)—extracted ion chromatograms of crude extract of *S. albus* I7act showing the production of the compounds **1**, **2**, **3**, **4**, **5**, and **6**, respectively. (**B**,**D**,**F**,**H**,**J**,**L**)—extracted ion chromatograms of crude extract of the negative control strain *S. albus* Del14. Compounds **1** to **6** cannot be detected in the extract of the control strain. The extracted mass ranges are shown on each single chromatogram.

To obtain the product of the type III PKS encoded by the *losA* gene, the latter was transcriptionally activated. The promoter cassette was PCR amplified and inserted in front of the *losA* gene by Red/ET [12,16]. In the obtained BAC I7act, the *losA* gene was set under the control of the strong synthetic promoter TS61 [17]. The constructed BAC was transferred into the *S. albus* Del14 strain by conjugation and the secondary metabolite profile of the obtained *S. albus* I7act strain was analyzed using LC-MS. This analysis revealed a number of new compounds in the extract of *S. albus* I7act which could not be observed in the extract of the control strain *S. albus* Del14 (Figure 2). Six most abundant compounds were the object of this study. For the sake of simplicity these compounds will be designated in the text by the numbers **1** to **6**. Besides UV/Vis detection, the compounds were analyzed with high-resolution mass spectrometry. Dissolved in methanol the compounds show a λ_max_ of 272 nm (Figure S1) and their experimental monoisotopic masses were calculated as follows: 336.2656 Da (compound **1**), 336.2657 Da (compound **2**), 334.2502 Da (compound **3**), 334.2497 Da (compound **4**), 348.2651 Da (compound **5**), and 362.2813 Da (compound **6**). The following molecular formulas were determined for the compounds **1** to **6**: C_21_H_36_O_3_ (compound **1**), C_21_H_36_O_3_ (compound **2**), C_21_H_34_O_3_ (compound **3**), C_21_H_34_O_3_ (compound **4**), C_22_H_36_O_3_ (compound **5**), and C_23_H_38_O_3_ (compound **6**) (Figure S2). The differences in the identified monoisotopic masses as well as the retention times of the compounds **1** to **6** indicate that they are probably structurally related. The pairs of the compounds **1**, **2** and **3**, **4** have similar monoisotopic masses, meaning that they might be constitutional isomers. The mass difference of 2 Da between the groups of compounds **1**, **2** and **3**, **4** implies that the corresponding compounds differ from each other in saturation grade of the alkyl chain. The mass difference of 14 Da between compound **5** and the group of compounds **3** and **4** suggests that the former contains one additional methylene group compared to the latter compounds. Similarly, compound **6** likely contains an additional methylene group compared to **5**.

The identified high-resolution monoisotopic masses of the compounds **1** to **6** were used for a search in the Dictionary of Natural Products (DNP) database of natural products. This search did not generate any matches. Further, the metabolic profiling of the wild type strain *M. endolithica* did not reveal the mass signals of compounds **1** to **6**. We therefore assumed that the identified compounds **1** to **6** might be new and heterologous expression facilitates their production under laboratory conditions.

### 2.2. Purification and Structure Elucidation

The results of the high-resolution LC-MS analysis and database surveys indicate that the compounds identified after activation of the *losA* gene might be new. In order to obtain structural information about the detected compounds, we set out to purify them. For this purpose, the *S. albus* I7act strain was inoculated into 10 L of the production medium. The biosynthetic products were extracted from the culture broth with ethyl acetate and the organic solvent was evaporated under vacuum. The compounds **1** to **6** were purified using size-exclusion and reverse phase chromatography (Figure S3). Only the compounds **1** and **2** with the determined monoisotopic masses of 336.2656 Da and 336.2657 Da were isolated in quantities sufficient for structure elucidation by NMR spectroscopy (approximately 1 mg). The other four compounds (**3**, **4, 5**, and **6**) were purified in submilligram quantities which were not sufficient to perform structure elucidation by NMR. All of the isolated compounds showed yellowish color and oily consistency.

The structure elucidation of compounds **1** and **2** was performed using nuclear magnet resonance spectroscopy (^1^H-NMR, HSQCed, HMBC, ^1^H-^1^H-COSY, and ^13^C-NMR) (Figure 3). All observed chemical shifts and correlations are presented in Table 2 (Figures S4–S11).

**Figure 3:**
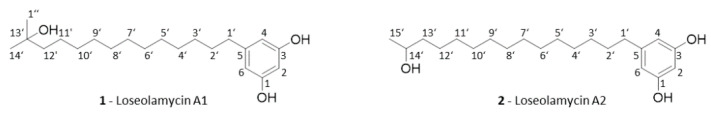
Structures of the loseolamycins A1 and A2 with numbered atoms according to the NMR data.

The ^1^H-NMR spectrum of the most abundant compound **1** in DMSO-*d*_6_ showed signals for a 5-substituted *m*-dihydroxyphenol (δ_H_ 5.98, s, H-2; δ_H_ 5.99, s, 2H, H-4 and H-6; δ_H_ 9.01, s, 2H, 1-OH, and 3-OH) as well as for saturated long alkyl chain (δ_H_ 1.15–1.30, H-2′–H-12′). The singlet methyl at 1.03 ppm integrating to six protons together with the signal for a tertiary alcohol (δ_C_ 68.70, C-13′) in the ^13^C-NMR spectrum gave hint to a terminal isopropyl alcohol moiety indicating a branched fatty alcohol. HMBC correlations (Table 2) supported these findings and led to the structure of the alkylresorcinol 5-(13-hydroxy-13-methyltetradecyl)benzene-1,3-diol, which was named loseolamycin A1 (compound **1**) (Figure 3).

Due to the low amount available, the structure elucidation of compound **2** with the monoisotopic mass of 336.2657 Da is solely based on the ^1^H-NMR and ^1^H-^1^H-COSY experiments and on the calculated molecular formula (Table 2). The obtained signals of the *m*-dihydroxyphenol and the corresponding signals of the alkyl chain are identical to those of **1**. Therefore, the sole difference between loseolamycin A1 and the compound **2** is the terminal group of the fatty alcohol. The chemical shift of the methyl group of compound **2** increased slightly when compared to the dimethyl moiety of loseolamycin A1. Therefore, its signal was then located within the broad fatty acid proton peak between 1.15 and 1.34 ppm, when measured in *d*_6_-DMSO. To circumvent this problem, a ^1^H-NMR and a ^1^H-^1^H-COSY was recorded in CDCl_3_. In the proton spectrum, a clear doublet becomes visible at 1.49 ppm, which shows a correlation in the COSY experiment to the signal of H-14′ at 4.35 ppm. The results of the NMR analysis and the observed monoisotopic mass allow for elucidation of compound **2** (5-(14-hydroxypentadecyl)benzene-1,3-diol). The compound **2** was named loseolamycin A2 (Figure 3). The determination of the optical rotation was not possible, because the substance had previously decomposed. The stereochemistry of the secondary alcohol C-14′ of loseolamycin A2 remains unsolved.

The compounds **3**, **4**, **5**, and **6** were purified in submilligram quantities which were not sufficient to perform NMR analysis. The determined monoisotopic masses and the calculated molecular formulas indicate that the compounds **3**, **4**, **5**, and **6** differ from loseolamycins A1 and A2 by saturation grade and the length of the alkyl chain (Figure S12).

### 2.3. Biosynthetic Scheme

**Figure 4:**
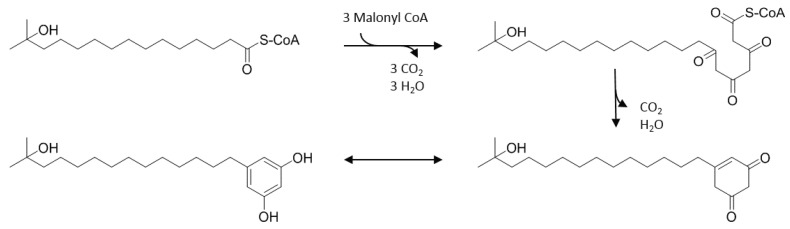
Proposed biosynthesis exemplarily for loseolamycin A1.

Alkylresorcinols as a product of bacterial type III PKS were mechanistically investigated and described before [18]. We propose that the activated C16 to C18 hydroxy fatty acids from the precursor pool of *S. albus* Del14 serve as starter units for the biosynthesis of loseolamycins. The type III PKS encoded by the gene *losA* catalyzes the decarboxylative condensation of three malonyl-CoA units with the activated starter unit. The decarboxylative aldol cyclization of the nascent polyketide chain as well as its tautomerism lead to the favorable resorcinol form of the final product (Figure 4) [19].

Bacterial type III PKS are often characterized by the lack of substrate specificity for the fatty acid starter unit [20,21,22]. Such promiscuity of the *losA* product might explain the production of numerous loseolamycin derivatives which differ in the length of their alkyl chain or its saturation state.

### 2.4. Biological Activity

Biological activity studies have been performed for the isolated loseolamycins. Agar disc diffusion tests with the test cultures *Escherichia coli*, *Pseudomonas putida*, and *Bacillus subtilis* were carried out to analyze if the compounds possess any antibacterial activity. No growth inhibition of *E. coli* and *P. putida* could be observed, indicating that loseolamycins A1 and A2 are not active against gram-negative bacteria. The compounds successfully inhibited the growth of the gram-positive bacterium *B. subtilis* (Figure S13). Additionally, loseolamycins were tested for herbicidal activity against the weed *Agrostis stolonifera*. Due to insufficient amounts of isolated individual loseolamycins, the prepurified fraction containing a mixture of loseolamycins with loseolamycin A1 as a major compound was used for the bioassay. Loseolamycins reproducibly demonstrated herbicidal activity by suppressing the germination of the *A. stolonifera* seeds (Figure S13). Further experiments are required to assess the activity of single derivatives and to propose a mode of action.

## 3. Materials and Methods

### 3.1. General Experimental Procedures

The strains, BACs and plasmids used in this work are listed in Table S1. The *Streptomyces* strains *S. albus* Del14, *S. albus* I7, and *S. albus* I7act were grown on soy flour mannitol (MS) agar [23] and in liquid tryptic soy broth (TSB; Sigma-Aldrich, St. Louis, MO, USA). Liquid DNPM medium (40 g/L dextrin, 7.5 g/L soytone, 5 g/L baking yeast, and 21 g/L MOPS, pH 6.8 as aqueous solution) was used for metabolite expression. DNPM as solid and liquid medium was used for cultivation of *Micromonospora endolithica*. *Bacillus subtilis*, *Pseudomonas putida*, and *Escherichia coli* strains were cultured in lysogeny broth (LB) medium [24]. The antibiotics kanamycin, apramycin, ampicillin, and nalidixic acid were added when required. Plant medium was prepared as aqueous solution containing 2.2 g/L Murashige and Skoog and 1.6 g/L Gamborg’s B5 plant salts.

### 3.2. Isolation and Manipulation of DNA

DNA manipulation, transformation into *E. coli* as well as intergeneric conjugation between *E. coli* and *Streptomyces* were performed according to standard protocols [23,24,25]. BAC DNA from the constructed genomic library IG652BAC1-2 of *Micromonospora endolithica* was isolated with the BACMAX™ DNA purification kit (Lucigen, Middleton, WI, USA). Promoter TS61 was inserted on the BAC I7 using Red/ET [26]. A respective gene cassette from pUC19 plasmid harboring an ampicillin resistance was amplified by PCR. PCR primers 20180710_02_fw and 20180710_02_rev were constructed with overhang regions complementary to the DNA bases in front of *losA* gene for site specific introduction of the cassette. For control of the successful recombination, restriction mapping as well as sequencing were performed. The restriction enzymes were provided by ThermoFisher Scientific (Waltham, MA, USA) or New England BioLabs NEB (Ipswich, MA, USA) and used as outlined in the instruction manuals.

### 3.3. Metabolite Extraction

For metabolite extraction, *Streptomyces* strains were grown in 15 mL of TSB in a 100 mL baffled flask for 1 day, and 1 mL of seed culture was used to inoculate 100 mL of DNPM production medium in a 500 mL baffled flask. Cultures were grown for 7 to 8 days at 28 °C and 180 rpm in an Infors multitron shaker (Infors AG, Basel, Switzerland). Metabolites were extracted from the culture supernatant with an equal amount of ethyl acetate, evaporated at 40 °C and kept at storage condition at 4 °C. The strain *S. albus* I7act was used for 10-L scale and the above described procedures were carried out accordingly using 100 single flasks for main culture.

### 3.4. Mass Spectrometry (MS) Metabolite Analysis

Dry sample extracts were dissolved in methanol prior to MS analysis. Sample compounds were separated on a Dionex Ultimate 3000 UPLC system (ThermoFisher Scientific, Waltham, MA, USA) equipped with an ACQUITY BEH C18 column 1.7 µm, 2.1 mm × 30 or 50 or 100 mm (Waters Corporation, Milford, MA, USA). A linear gradient from hydrophilic to lipophilic mobile phase was run as follows: [A] water + 0.1% formic acid/[B] acetonitrile + 0.1% formic acid, 5% to 95% [B] at flow rate of 0.6 mL/min. Behind the PDA detector, an amaZon speed or LTQ Orbitrap XL mass spectrometer (Bruker, Billerica, MA, USA) were coupled to the system. LTQ Orbitrap XL provides a mass accuracy of 5 ppm. Mass analysis was performed using positive or negative ionization and a mass range selection of *m*/*z* 200 to 2000. In positive mode, loss of water occurs during ionization of most loseolamycin derivatives. Mass data was analyzed using the softwares Compass Data Analysis 4.1 and Xcalibur 3.

### 3.5. Purification

The crude extract from the 10-L culture of recombinant strain *S. albus* I7act was dissolved in methanol. Concentrated extract solution was loaded onto a LC column for a first purification step using size exclusion (SEC) as separation mode. The column was packed with a stationary phase of Sephadex-LH20 and run with an isocrate of pure methanol. Eluates were checked on mass spectrometer for content and purity of loseolamycin derivatives and pooled to prepurified loseolamycin fractions. Pooled fractions were dried at 40 °C and stored in the refrigerator at 4 °C. For separation of loseolamycin derivatives, a second chromatographic step was performed. The fraction pool was redissolved in methanol and applied on a reversed phase (RP). An Agilent Infinity 1200 series HPLC system equipped with Synergi^TM^ 4 µm Fusion-RP 80 Å 250 × 10 column (phenomenex, Torrance, CA, USA) was used and elution was carried out as follows: linear gradient of [A] water + 0.1% formic acid/[B] acetonitrile + 0.1% formic acid, 50% to 95% [B] in 20 min at flow rate of 4 mL/min and column oven temperature 45 °C. Fractions were controlled with HPLC-MS before final pooling to the six pure isolates **1** to **6**.

### 3.6. Nuclear Magnetic Resonance (NMR) Spectroscopy

The isolated compounds were dissolved in 300 μL deuterated solvent (DMSO-*d*_6_, MEOD-*d*_4_, CDCl_3_) and measured in a corresponding 5-mm Shigemi-tube (DEUTERO GmbH, Kastellaun, Germany). NMR data (^1^H, HH-COSY; TOCSY; HMBC, HSQC, ^13^C) were acquired with standard pulse programs on either a Bruker Ascend 700 spectrometer equipped with a 5-mm TXA Cryoprobe at 300K or a Bruker Avance 500 spectrometer equipped with a 5-mm BBO Probe (Bruker BioSpin GmbH, Rheinstetten, Germany). Bruker TopSpin 3.5a software was used for the evaluation of NMR results.

### 3.7. Activity Testing

For testing of antibacterial activity, filter discs were impregnated with methanolic solutions of pure loseolamycins A1 and A2 and dried for 20 min. Meanwhile a concentrated cell suspension of each *E. coli*, *B. subtilis* and *P. putida*, was mixed with 1 mL of LB soft agar and poured onto a thin LB agar plate. The dried filter discs were placed onto the prepared plates and incubated at 29 °C for 15 h. Visual evaluation of the inhibition zone was performed against a methanol negative control which was processed as described before for the sample disc.

The herbicidal activity assay against the weed *A. stolonifera* was performed in 96-well plates. Serial dilutions of the sample containing loseolamycins were performed in the plant medium reducing the concentration of the compound by a factor of 0.5 each. 10 seeds of the test plant were put in each well containing 195 µL of medium and cultivated in a greenhouse with fluorescent light for 6 days at room temperature. As a control, dilutions of methanol in plant medium were used. The number of germinated seedlings was determined by visual analysis.

### 3.8. Genome Mining and Bioinformatic Analysis

The genome of *M. endolithica* was screened for secondary metabolite biosynthetic gene clusters using the antiSMASH online tool (https://antismash.secondarymetabolites.org/#!/start) [13]. Analysis of genetic data was performed using the Geneious software, version 11.0.3 [27]. The genomic sequence of BAC I7 was deposited in GenBank under accession number MT904273. For dereplication, the Dictionary of Natural Products (DNP) 28.1 was used as database of known natural products.

## 4. Conclusions

The production of secondary metabolites from actinobacteria strongly depends on the bacterial needs and many gene clusters remain silent under laboratory working conditions. Heterologous expression of a promoter activated type III PKS gene of *Micromonospora endolithica* facilitated the production of structurally new bacterial alkylresorcinols that we called loseolamycins. Unusual hydroxylated C16 to C18 fatty acids from the host’s primary metabolism are utilized as starter units. The here isolated loseolamycins add further structural diversity to the class of alkylresorcinols. Furthermore, the new compounds exhibit herbicidal and antibacterial activity that might drive future research efforts.

## Figures and Tables

**Table 1:** Proposed functions of the genes in the chromosomal fragment cloned in BAC I7.

Gene	Identifier	Putative Function
1	*LU17765_001550*	l-aspartate oxidase
2	*LU17765_001560*	carboxylating nicotinate-nucleotide diphosphorylase
3	*LU17765_001570*	Type III pantothenate kinase
4	*LU17765_001580*	putative methyltransferase
5	*LU17765_001590*	putative methyltransferase
6	*LU17765_001600*	Lysine-tRNA ligase
7	*LU17765_001610*	Nucleoid-associated protein Lsr2
8	*LU17765_001620*	ATP-dependent Clp protease ATP-binding subunit
9	*LU17765_001630*	A/G-specific adenine glycosylase
10	*LU17765_001640*	ACT domain-containing protein
11	*LU17765_001650*	Peptide deformylase
12	*LU17765_001660*	Hypothetical protein
13	*LU17765_001670*	DNA integrity scanning protein DisA
14	*LU17765_001680*	DNA repair protein RadA
15	*LU17765_001690*	Hypothetical protein
16	*LU17765_001700*	UbiA family prenyltransferase
17	*LU17765_001710*	putative methyltransferase
18	*LU17765_001720*	acyl-CoA dehydrogenase
19 [*losA*]	*LU17765_001730*	type III polyketide synthase
20	*LU17765_001740*	Hypothetical protein
21	*LU17765_001750*	Hypothetical protein
22	*LU17765_001760*	CarD family transcriptional regulator
23	*LU17765_001770*	2-C-methyl-d-erythritol 4-phosphate cytidylyltransferase
24	*LU17765_001780*	2-C-methyl-d-erythritol 2,4-cyclodiphosphate synthase
25	*LU17765_001790*	tetratricopeptide repeat protein

**Table 2:** NMR Spectroscopic data (500 MHz, *d*_6_-DMSO) for loseolamycin A1 and loseolamycin A2.

1-Loseolamycin A1				2-Loseolamycin A2	
Position	δ_C_, Type	δ_H_, Type (*J* [Hz])	HMBC ^1^	Position	δ_H_, Type
1	158.14, C	-	-	1	-
2	99.91, CH	5.98, s	3, 4, 6	2	5.98, m
3	158.14, C	-	-	3	-
4	106.24, CH	5.99, s	3, 6, 1′	4	5.98, m
5	144.15, C	-	-	5	-
6	106.24, CH	5.99, s	3, 4, 1′	6	5.98, m
1′	35.24, CH2	2.33, dd, 7.5 Hz	5, 4, 6, 2′, 8′	1′	2.32, m
2′	30.67, CH2	1.45, m	-	2′	1.44, m
3′	29.77, CH2	1.15–1.24, m	-	3′	1.15–1.34, m
4′	29.10, CH2	1.15–1.24, m	-	4′	1.15–1.34, m
5′	29.02, CH2	1.15–1.24, m	-	5′	1.15–1.34, m
6′	29.02, CH2	1.15–1.24, m	-	6′	1.15–1.34, m
7′	29.01, CH2	1.15–1.24, m	-	7′	1.15–1.34, m
8′	28.99, CH2	1.15–1.24, m	-	8′	1.15–1.34, m
9′	28.87, CH2	1.15–1.24, m	-	9′	1.15–1.34, m
10′	28.64, CH2	1.15–1.24, m	-	10′	1.15–1.34, m
11′	23.83, CH2	1.26, m	9′, 14′, 1″, 8′, 12′, 13′	11′	1.15–1.34, m
12′	43.67, CH2	1.29, m	13′, 14′, 1″, 10′	12′	1.44, m
13′	68.70, C	-	-	13′	1.52, m
14′	29.25, CH3	1.03, s	13′, 12′, 1″, 11′	14′	4.35, m
1″	29.25, CH3	1.03, s	13′, 12′, 14′, 11′	15′	1.49, d
1-OH	-	9.01, s br	-	1-OH	9.06, s br
3-OH	-	9.01, s br	-	3-OH	9.06, s br
13′-OH	-	4.01, s br	14′, 1″	14′-OH	5.30, s

^1^ HMBC correlations, optimized for 6 Hz, are from protons stated to the indicated carbon.

## Supplementary Materials

The following are available online: Figure S1: UV/Vis spectrum of loseolamycin A1. Figure S2: Mass spectra of loseolamycins. Figure S3: Purity of the isolated loseolamycin derivatives. Figure S4: ^1^H-NMR spectrum (500 MHz, DMSO-*d*_6_) of loseolamycin A1. Figure S5: ^1^H-^1^H-COSY spectrum (500 MHz, DMSO-*d*_6_) of loseolamycin A1. Figure S6: HSQC-spectrum (500 MHz; 125 MHz, DMSO-*d*_6_) of loseolamycin A1. Figure S7: HMBC-spectrum (500 MHz; 125 MHz, DMSO-*d*_6_) of loseolamycin A1. Figure S8: ^13^C-spectrum (125 MHz, DMSO-*d*_6_) of loseolamycin A1. Figure S9: ^1^H-NMR spectrum (500 MHz, DMSO-*d*_6_) of loseolamycin A2. Figure S10: ^1^H-NMR spectrum (500 MHz, CDCl_3_) of loseolamycin A2. Figure S11: ^1^H-^1^H-COSY (500 MHz, CDCl_3_) of loseolamycin A2. Figure S12: Proposed structures of loseolamycins B1, B2, C and D based on MS/MS experiments. Figure S13: Antibacterial and herbicidal activity of loseolamycins. Table S1: Organisms, BACs, plasmids and primer used in this work.
