# Supplementary material for: Loseolamycins: A Group of New Bioactive Alkylresorcinols Produced after Heterologous Expression of a Type III PKS from Micromonospora endolithica
Source: Molecules. 2020 Oct 9;25(20):4594. doi: 10.3390/molecules25204594 (PMC7587189; doi:10.3390/molecules25204594)
Supplement: Supplementary file 1 [file molecules-25-04594-s001.pdf]

Article

Loseolamycins: a group of new bioactive alkylresorcinols produced after heterologous expression of a type III PKS from *Micromonospora endolithica*

Constanze Lasch [1], Nils Gummerlich [1], Maksym Myronovskyi [1], Anja Palusczak [1], Josef Zapp [2] and Andriy Luzhetskyy [1,3,*]

[1] Pharmazeutische Biotechnologie, Universität des Saarlandes, Saarbrücken, Germany; constanze.lasch@uni-saarland.de (C.L.), nils.gummerlich@gmx.de (N.G.); maksym.myronovskyi@uni-saarland.de (M.M.); anja.palusczak@uni-saarland.de (A.P.); a.luzhetskyy@mx.uni-saarland.de (A.L.)

[2] Pharmazeutische Biologie, Universität des Saarlandes, Saarbrücken, Germany; j.zapp@mx.uni-saarland.de (J.Z.)

[3] Helmholtz-Institut für Pharmazeutische Forschung Saarland, Saarbrücken, Germany

* Correspondence: a.luzhetskyy@mx.uni-saarland.de; +49 681 302 70200 (A.L.)

Received: date; Accepted: date; Published: date

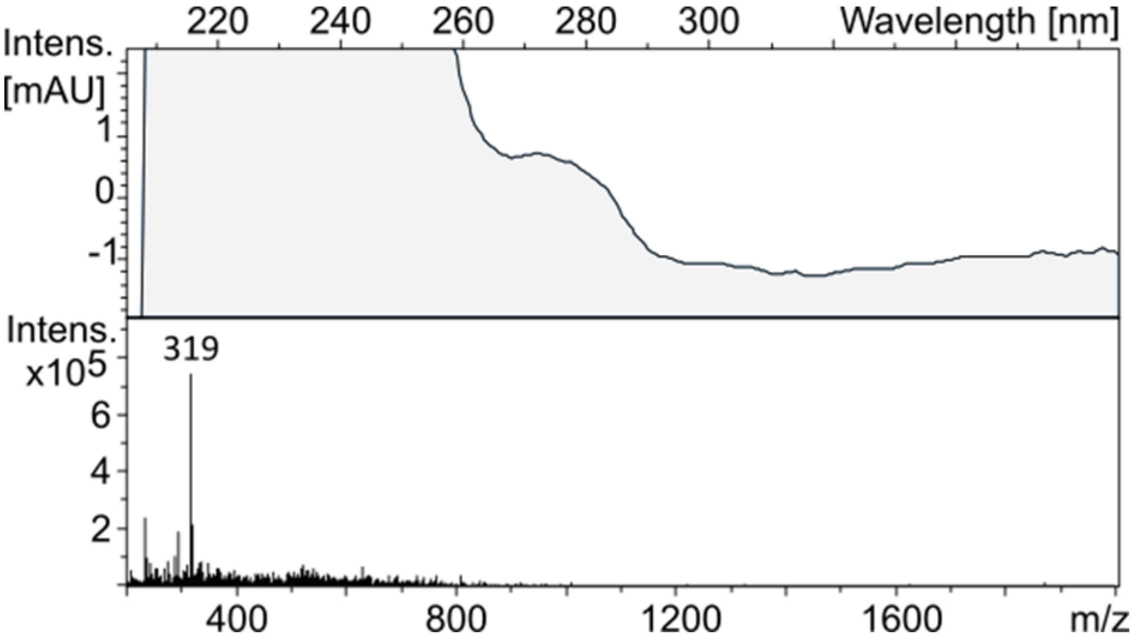

Figure S1. *UV/Vis spectrum of loseolamycin A1.*

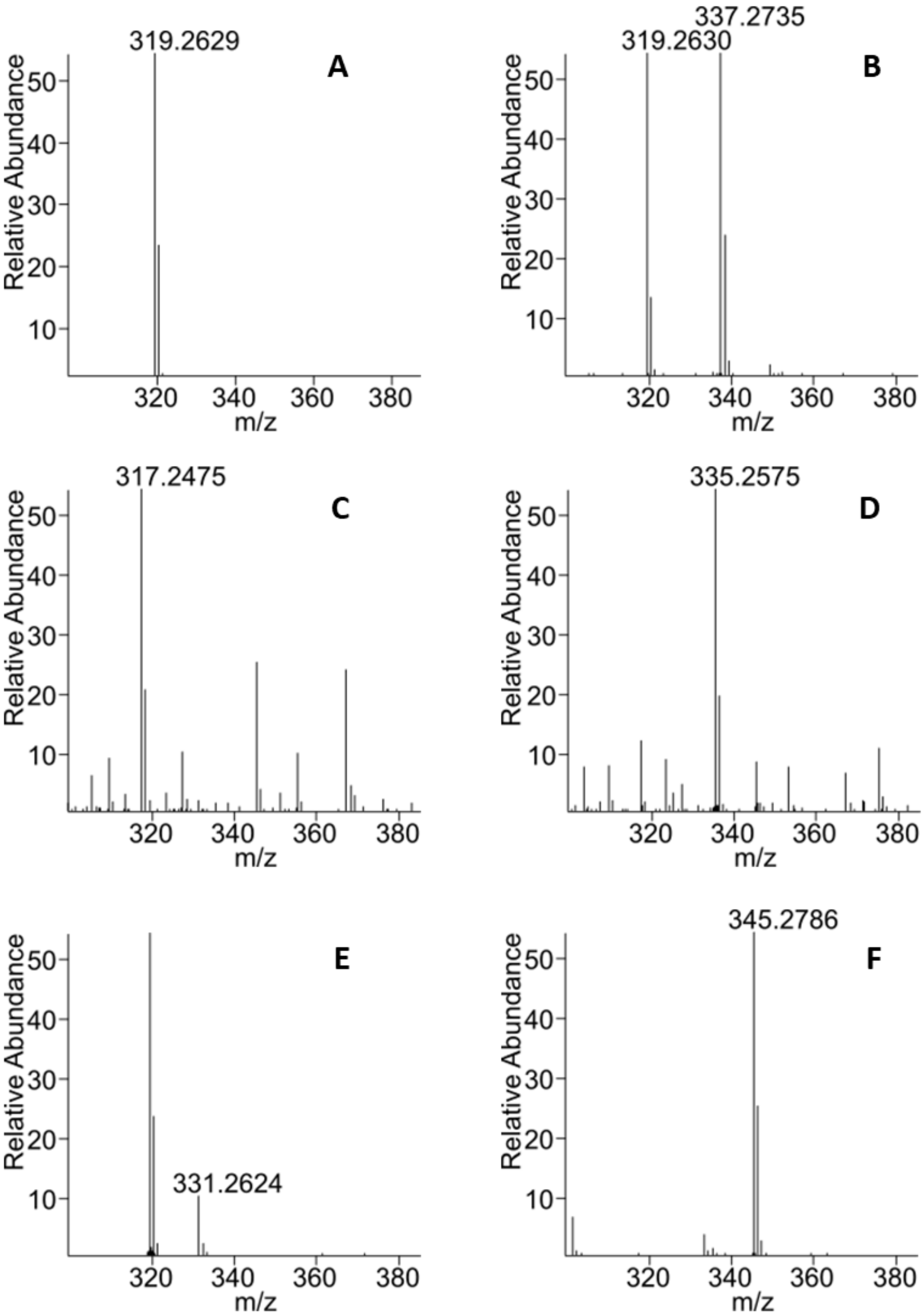

Figure S2. *Mass spectra of loseolamycins using positive ionization mode. A - compound **1**, B – compound **2**, C – compound **3**, D – compound **4**, E – compound **5**, F – compound **6**.*

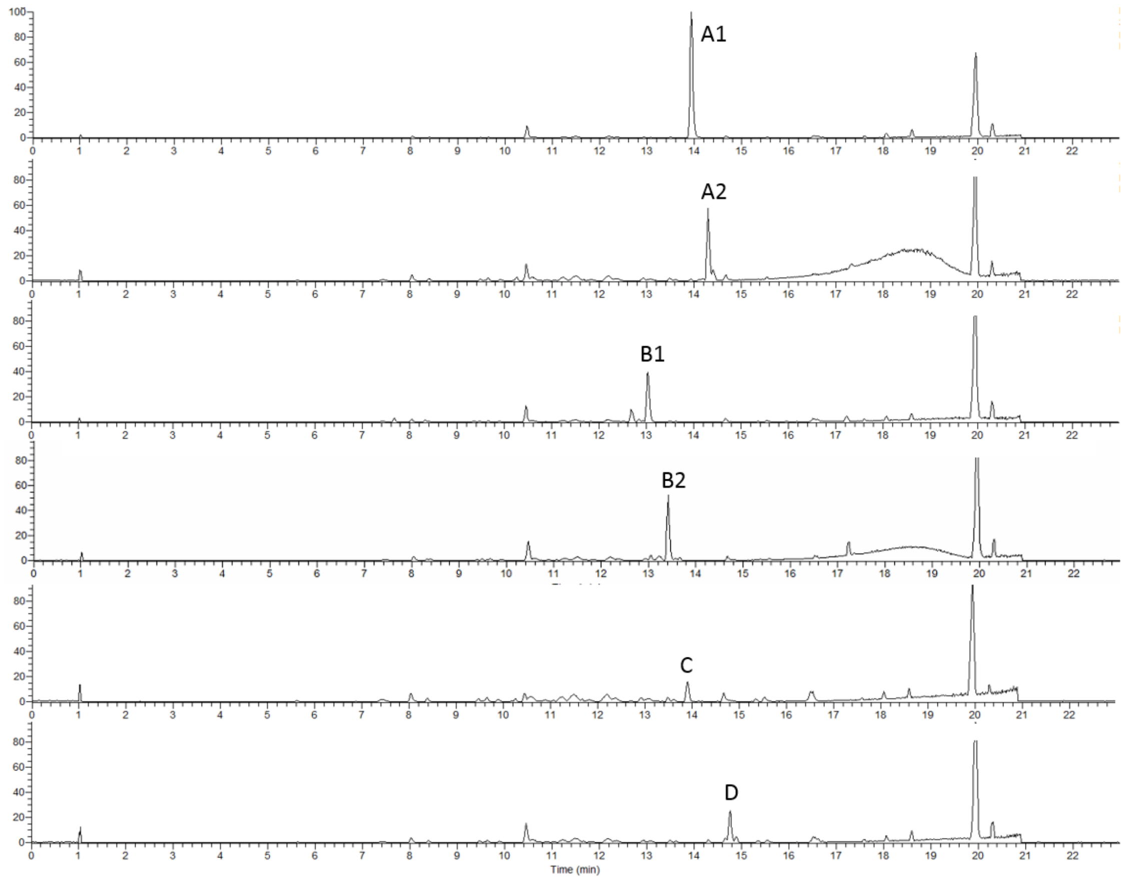

Figure S3. *Purity of the isolated loseolamycin derivatives A1 (compound **1**), A2 (compound **2**), B1 (compound **3**), B2 (compound **4**), C (compound **5**) and D (compound **6**) after two chromatographic steps. Base Peak Chromatograms (BPC) are shown.*

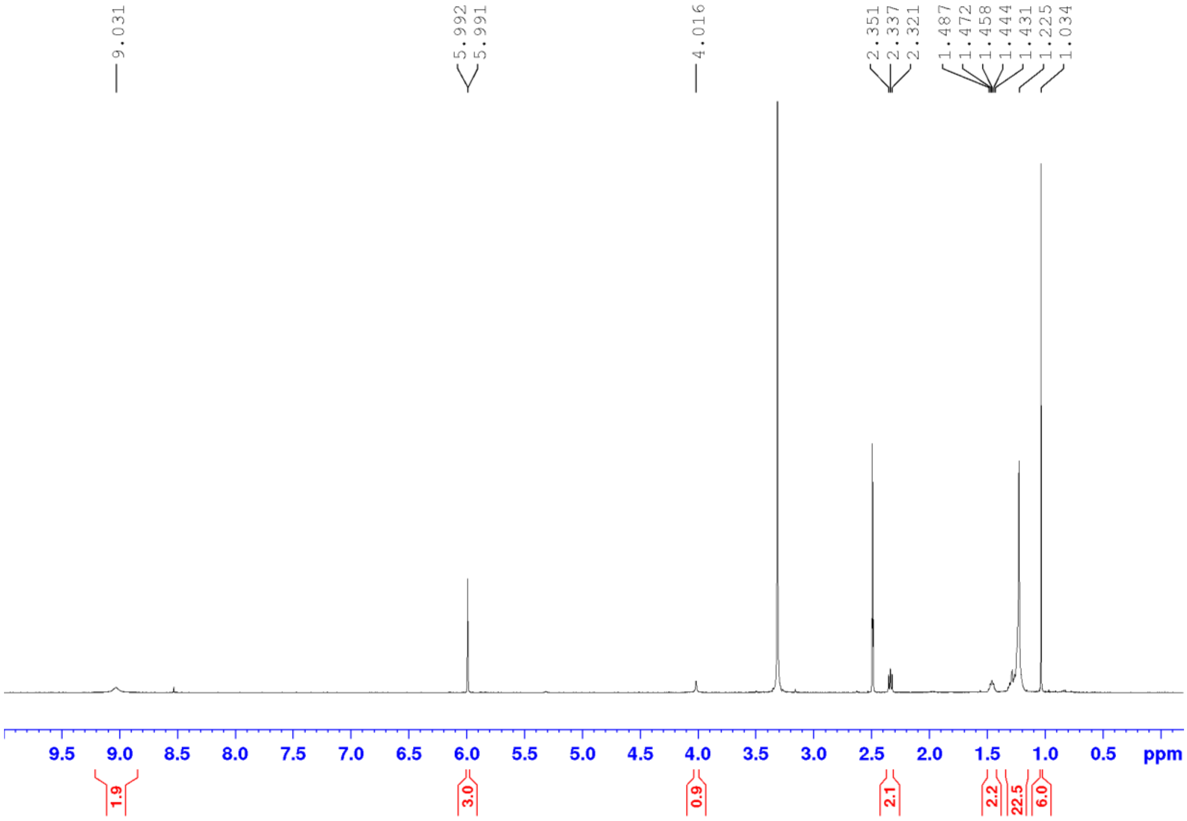

Figure S4. *^{1}H-NMR spectrum (500 MHz, DMSO-d6) of loseolamycin A1, complete spectrum.*

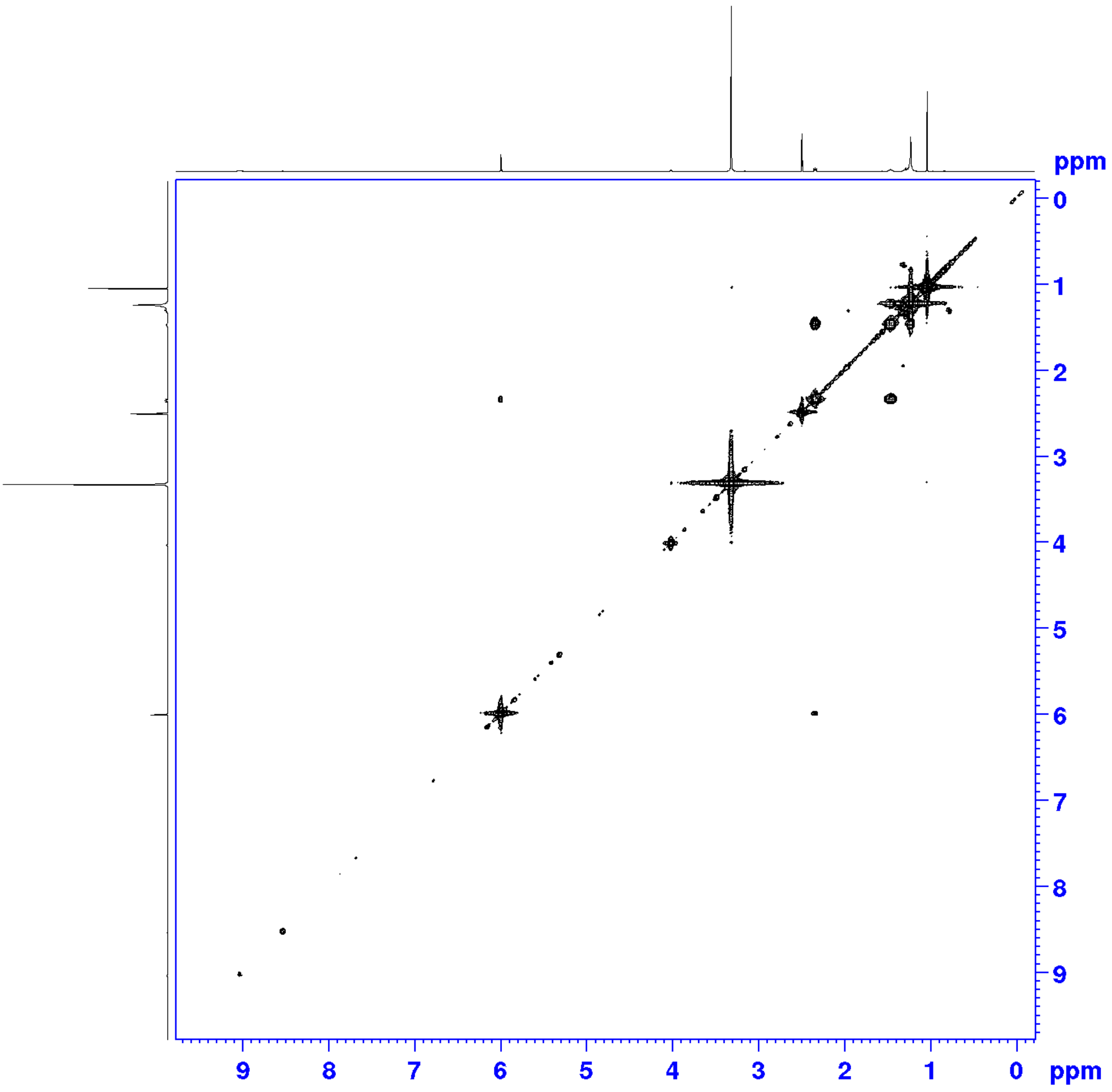

Figure S5. *^{1}H-^{1}H-COSY spectrum (500 MHz, DMSO-d6) of loseolamycin A1, complete spectrum.*

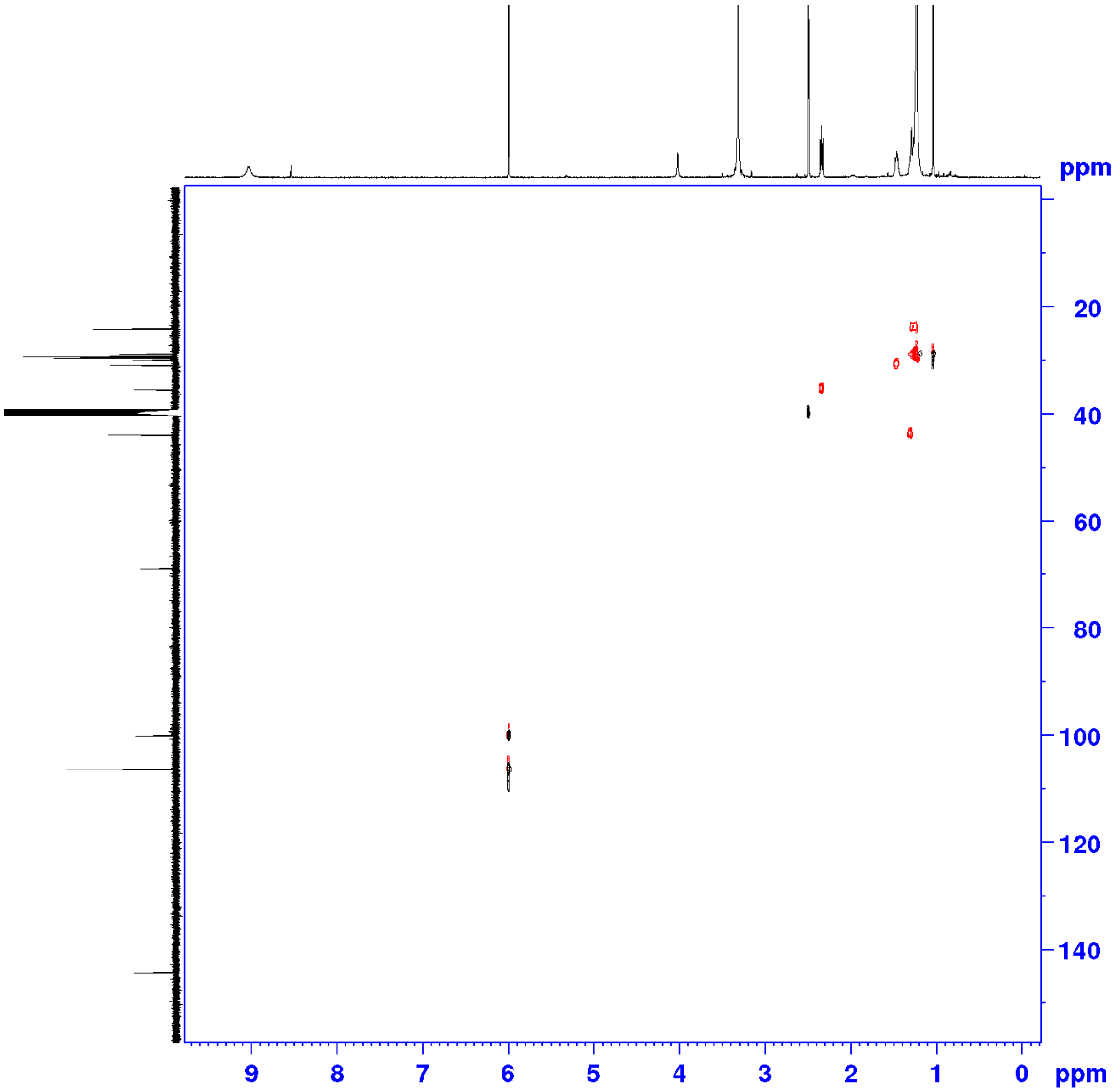

34

35 ***Figure S6.*** *HSQC-spectrum (500 MHz; 125 MHz, DMSO-d6) of loseolamycin A1, complete spectrum.*

36

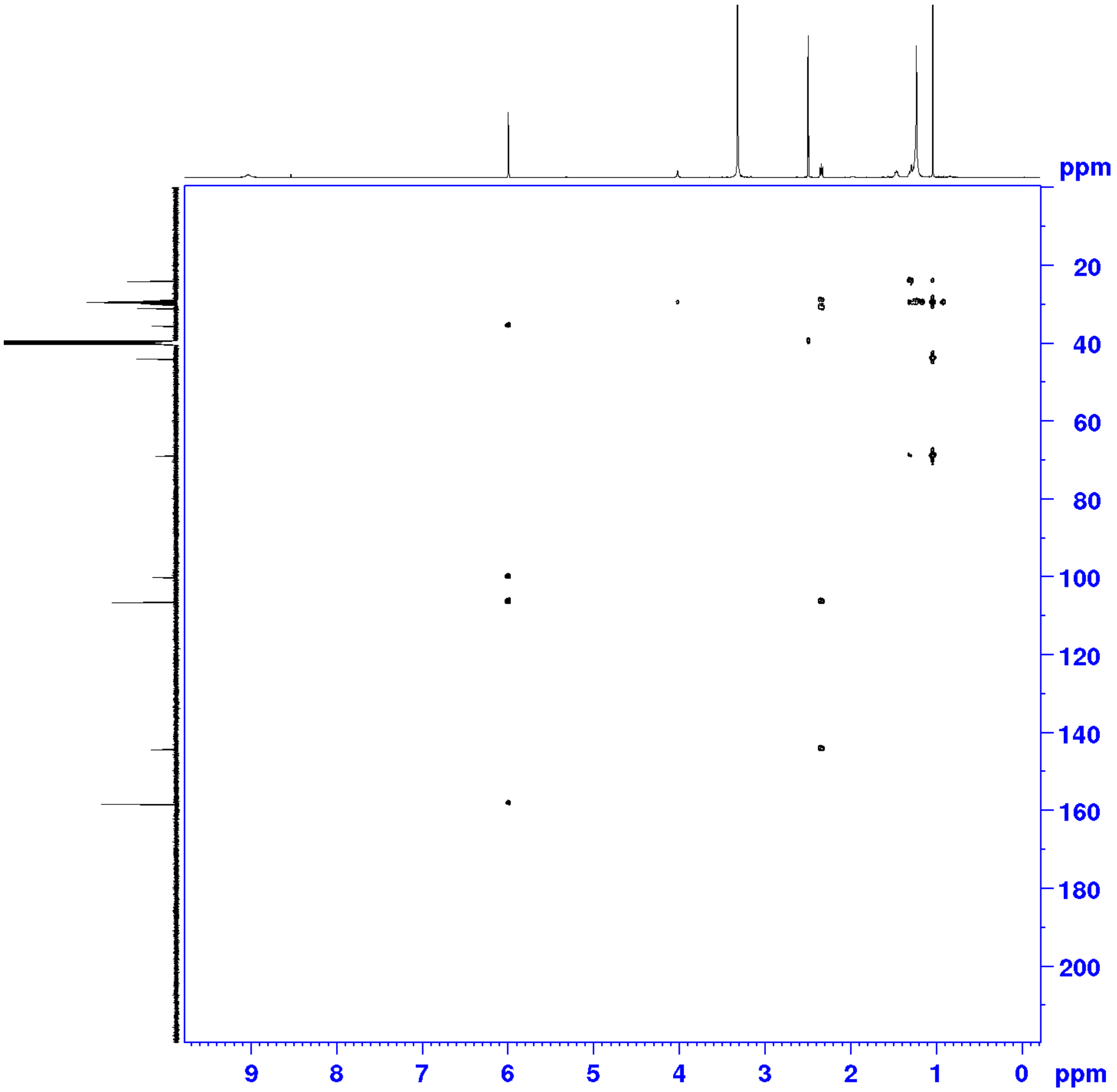

37

38 ***Figure S7.*** *HMBC-spectrum (500 MHz; 125 MHz, DMSO-d6) of loseolamycin A1, complete spectrum.*

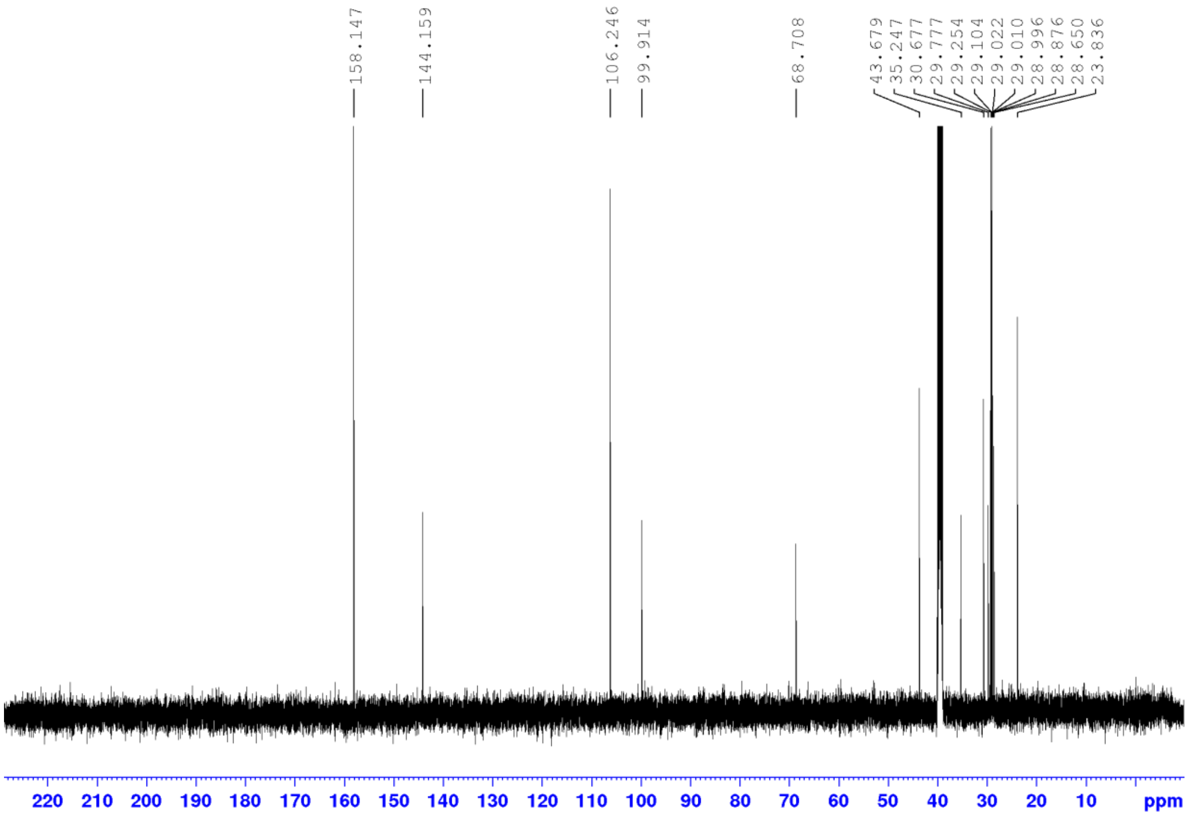

Figure S8. *^{13}C-spectrum (125 MHz, DMSO-d6) of loseolamycin A1, complete spectrum.*

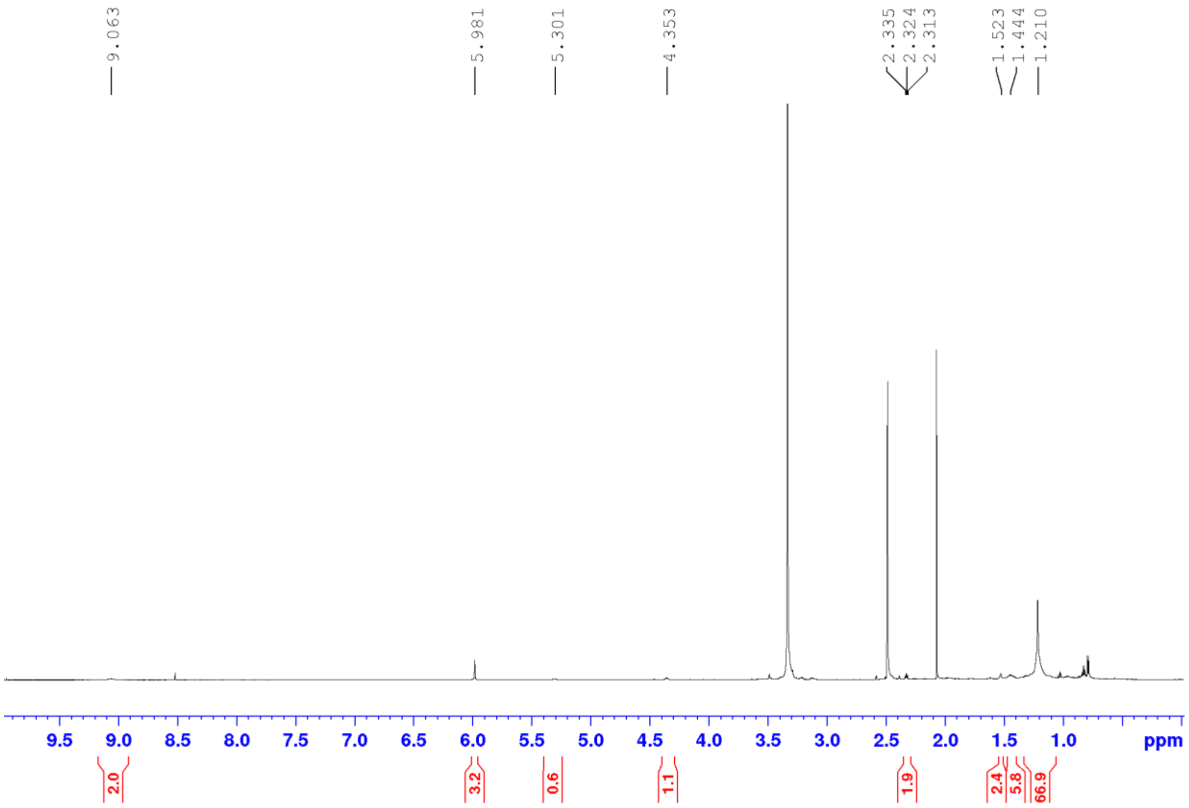

Figure S9. *^{1}H-NMR spectrum (500 MHz, DMSO-d6) of loseolamycin A2, complete spectrum.*

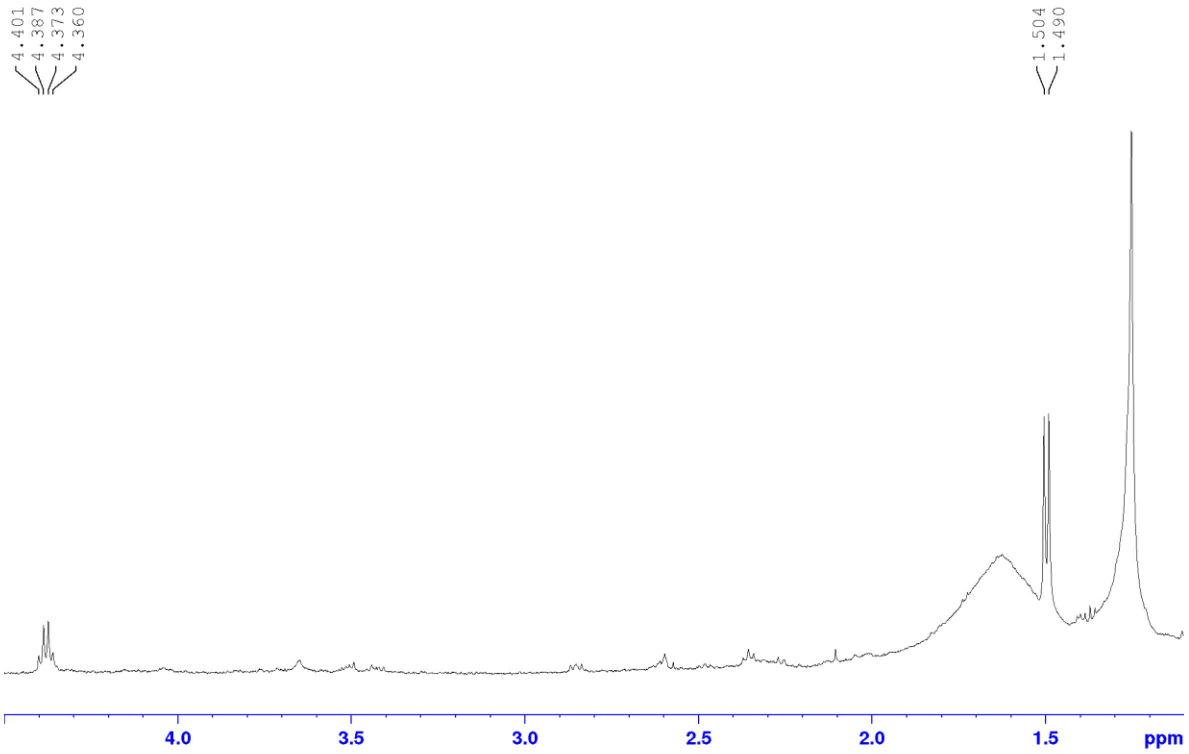

45

46 ***Figure S10.*** *^{1}H-NMR spectrum (500 MHz, CDCl3) of loseolamycin A2, zoomed from 1.0 – 4.5 ppm.*

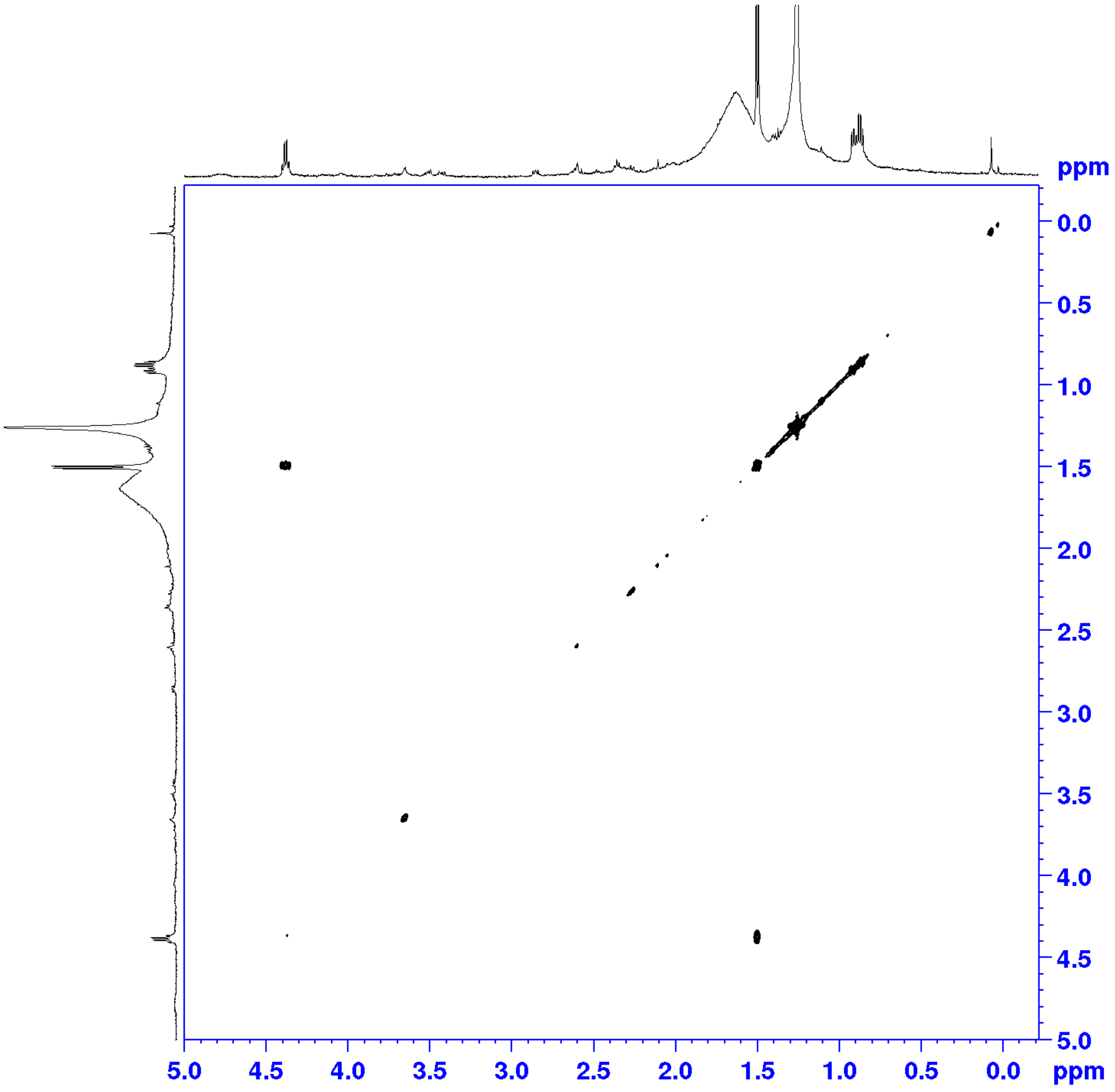

Figure S11. *^{1}H-^{1}H -COSY (500 MHz, $CDCl_3$) of loseolamycin A2, zoomed from 1.0 – 4.5 ppm.*

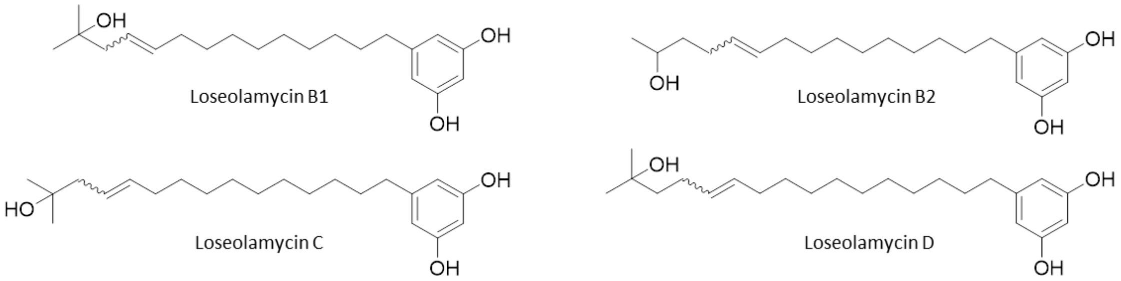

Figure S12. *Proposed structures of loseolamycins B1 (compound **3**), B2 (compound **4**), C (compound **5**) and D (compound **6**) based on MS/MS experiments.*

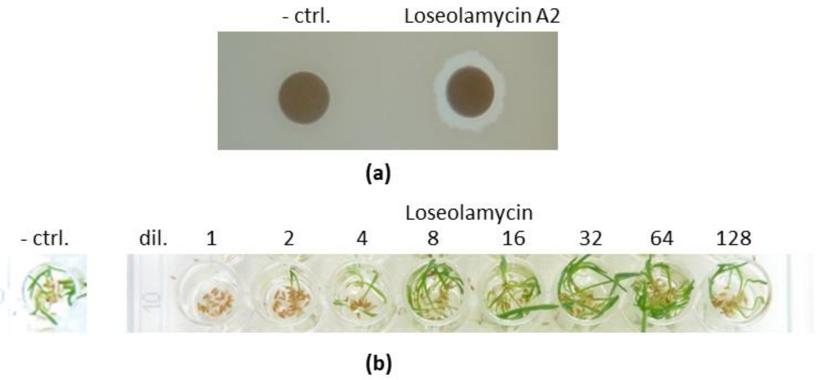

Figure S13. *Antibacterial and herbicidal activity. (a) Loseolamycin A2 on a filter disc inhibits the growth of B. subtilis. The sample's solvent methanol was used as negative control (- ctrl) and shows no inhibition zone. (b) A mixture of loseolamycin derivatives inhibits germination of the weed Agrostis stolonifera. The phytotoxic effect was reproduced once and is concentration dependent. Methanol in plant medium was used as negative control (- ctrl) and shows no inhibition of seed germination.*

Table S1. *Organisms, BACs, plasmids and primer used in this work.*

**Material**	**Purpose**
**A. Organisms**	
Micromonospora endolithica LU17765	originating strain of type III PKS [BASF]
Streptomyces albus Del14	optimized heterologous host [1]
Escherichia coli GB05 RedCC	cloning host [Helmholtz-Institut für Pharmazeutische Forschung Saarland (HIPS)]
Escherichia coli ET12567 pUB307	alternate host intergeneric conjugation [2]
Escherichia coli GB 2005	bioactivity test
Pseudomonas putida KT2440	bioactivity test
Bacillus subtilis ATCC 6633	bioactivity test
Agrostis stolonifera	bioactivity test
**B. BACs**	
I7 [IG652BAC1-2]	heterologous expression of type III PKS [GenBank: MT904273]
I7act	heterologous expression of promoter activated type III PKS
**C. Plasmids**	
pUC19	promoter TS61 / ampicillin resistance marker
**D. PCR primer**	
20180710_02_fw [I7act]	TGAATCAGATTTGCGAGTCCCGCAGTCGCGAACGGACCGGACTCGTTGGTCGT CAGGTGGCACTTTTCG
20180710_02_rev [I7act]	CACCGGCACGCCCATGTCCCCCACCTCTCGTCCCCGATCCCCCACGCTTCGCGG ATATCCTACTATGCCGAGGTATAATGTAGCCAGCGTGTTACCAATGCTTAATCA GTG

REFERENCES

[1] M. Myronovskyi, B. Rosenkränzer, S. Nadmid, P. Pujic, P. Normand, A. Luzhetskyy, Generation of a cluster-free Streptomyces albus chassis strains for improved heterologous expression of secondary metabolite clusters, Metab. Eng. 49 (2018) 316–324. https://doi.org/10.1016/j.ymben.2018.09.004.

[2] F. Flett, V. Mersinias, C.P. Smith, High efficiency intergeneric conjugal transfer of plasmid DNA from Escherichia coli to methyl DNA-restricting streptomycetes, FEMS Microbiol. Lett. 155 (2006) 223–229. https://doi.org/10.1111/j.1574-6968.1997.tb13882.x.
